# Profiling of phytochemicals in *Adenophora triphylla* using LC-Q-TOF/MS-based untargeted metabolomics†

**DOI:** 10.1039/d5ra00186b

**Published:** 2025-06-02

**Authors:** Yoonjeong Kim, Jiye Pyeon, Yeong Eun Yu, Cheol-jong Jung, Do Sang Lee, Im-Joung La, Younghwa Kim

**Affiliations:** a Department of Food Science and Biotechnology, Kyungsung University Busan 48434 Republic of Korea younghwakim@ks.ac.kr; b Central Research Center, Okchundang Inc. Daegu 41059 Republic of Korea; c Atomy R&D Center Gongju 32511 Republic of Korea

## Abstract

*Adenophora triphylla* has been widely used in traditional oriental medicine and can be used in the functional food industry because of its health-beneficial properties. However, although previous studies have focused on specific bioactive compounds, such as saponins and flavonoids, a comprehensive understanding of the overall chemical composition of *A. triphylla* root and its processed extracts remains limited. To address this gap, this study applied a non-targeted metabolomics approach using LC-Q-TOF-MS to compare the metabolite profiles of *A. triphylla* root (AR) and its hydrothermal extract (ARE). A total of 23 and 15 compounds were tentatively identified from AR in negative and positive ion modes, while 19 and 9 compounds were detected from ARE, respectively. Although the classes of compounds were largely similar, the results of multivariate analyses, such as principal component analysis (PCA), partial least-squares discriminant analysis (PLS-DA), and orthogonal partial least-squares discriminant analysis (OPLS-DA), revealed distinct metabolic patterns between AR and ARE. PCA accounted for more than 95% of the total variance, and the *R*^2^*Y* and *Q*^2^ values of PLS-DA and OPLS-DA exceeded 0.9, clearly distinguishing AR from ARE. Thirty-two compounds were identified as differential metabolites with variable importance in projection scores of 1 or higher, and 21 compounds were identified in AR and ARE. This study provides novel insights into how hydrothermal extraction affects the overall metabolite composition of *A. triphylla*, supporting its potential application in the development of functional food ingredients.

## Abstract 2

This study investigated the metabolic changes upon hydrothermal extraction of *A. triphylla* root, employing untargeted metabolomics analysis.

## Graphical abstract

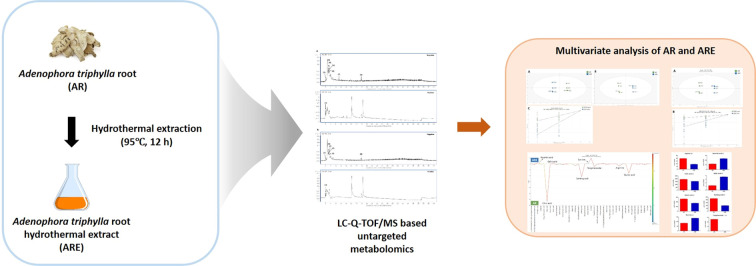

## 1 Introduction

Metabolomics is a discipline that investigates all metabolites produced by living organisms to understand biological processes or identify disease biomarkers. It is recognized as an important field in systems biology, along with genomics, transcriptomics, and proteomics.^1^ Untargeted metabolomics is a tool for understanding the overall metabolic pathway by examining all its metabolites, focusing on individual metabolites.^2^ It can be used to monitor metabolic changes and serves as an important tool for biological and physiological research.^3^ Metabolomics has emerged as a pivotal technique in biological study and food science research. This analytical approach has been widely used to study nutrient bioavailability and metabolic pathways by evaluating metabolite changes following food intake.^4,5^ Furthermore, metabolomics facilitates the investigation of chemical transformations that occur during food processing.^6,7^ Such analyses provide vital insights into determining optimal processing conditions and improving food quality and safety.^8^ Recently, liquid chromatography-quadrupole-time-of-flight mass spectrometry (LC-Q-TOF/MS) has been widely used in untargeted metabolomics research.^9,10^ LC-Q-TOF-MS provides high resolution and accuracy and the potential to precisely examine a wide range of chemicals in complex samples.^11,12^ Since the efficacy of the multicomponents in herbs originates from the interactions between complex components, traditional analytical approaches that target only specific active components make it difficult to fully understand this complexity.^13^ To overcome these limitations, untargeted metabolomics can be used to assess herb properties and follow overall metabolic changes to establish efficacy.^14^ This approach contributes to assessing the chemical composition of herbs, demonstrating their physiological effects and investigating new applications.^15,16^

*Adenophora triphylla* is an herbaceous perennial plant belonging to the Campanulaceae family.^17^ The dried roots of *A. triphylla* are called “sa-sam” in Korea and are eaten roasted like deodeok or balloon flower roots, or cooked in pickles, seasoning, stir-fried and other dishes.^18^ Furthermore, the dried root of *A. triphylla* is traditionally used as a medicine in Korea and China for the treatment of several diseases, such as chronic bronchitis and whooping cough.^19^ Recently, *A. triphylla* was shown to possess antibacterial, anticancer, anti-inflammatory, analgesic, and immunomodulatory activities.^20^ In addition, the water extracts of *A. triphylla* alleviate estrogen deficiency-related menopausal symptoms in postmenopausal women.^21^ Moreover, *A. triphylla* contains luteolin, chlorogenic acid, triterpenoid saponin and polyhydroxylated alkaloids, which exhibit a wide range of biological activities, including antimutagenic and antitumor activities, antioxidant, anticancer, and anti-inflammatory effects.^18,22,23,26,27^ Owing to these functional properties, it has attracted attention as an industrial material in the functional food, dietary supplement, and pharmaceutical industries.^30^ Previous studies on *A. triphylla* have primarily focused on specific classes of phytochemicals, such as saponins and flavonoids, or the particular biological activities of the plant.^31–33^ On the other hand, dried *A. triphylla* is traditionally ingested in the form of hydrothermal extracts, which are prepared by boiling the root in water. This method of preparation is not only aligned with traditional medicinal practices but is also widely used in modern functional food formulations. However, little is known about the broader chemical changes that occur during hydrothermal processing. To address this aspect, this study applied an untargeted metabolomics approach using LC-Q-TOF/MS to compare the metabolic profiles of dried *A. triphylla* root (AR) and its hydrothermal extract (ARE) to reveal how processing affects the plant's overall chemical composition. In addition, this study investigated processing-induced alterations in phytochemical composition and provided foundational insights for the further development of *A. triphylla* as a functional food ingredient.

## 2 Materials and methods

### 2.1 Plant materials

AR and ARE were obtained from the R&D center of Ockcheondang Co., Ltd. (Kyeongsan, Korea). The dried AR was imported from China and used as the raw material for extract preparation. ARE was prepared by extracting 1 kg of AR with 30 kg of water at 95 °C for 12 h, repeated twice to ensure thorough extraction. 50% dextrin was then added to the extract solution, and the mixture was spray-dried to obtain the final powdered extract.

### 2.2 Chemicals and reagents

Formic acid was purchased from Merck (Darmstadt, Germany), and high-performance liquid chromatography (HPLC)-grade acetonitrile (ACN) was purchased from Fisher Scientific (Seoul, Korea). HPLC-grade methanol and isopropanol (IPA) were supplied by Honeywell Burdick & Jackson (Honeywell Burdick & Jackson, Muskegon, MI, USA) and deionized water was used as a Milli-Q water purification system (Millipore Ltd., Bedford, MA, USA). The standards (purity ≥ 95%) for syringin, coumarin and syringic acid were purchased from Sigma-Aldrich (St. Louis, MO, USA) and used as external standards for the identification of compounds. Isotope-labeled L-[^13^C_1_] phenylalanine, which was used as an internal standard, was purchased from Sigma-Aldrich.

### 2.3 Untargeted metabolomics analysis of AR and ARE using LC-Q-TOF/MS

#### 2.3.1 Sample preparation

AR and ARE (1 g) were weighed into a 15 mL falcon tube and mixed with 3 mL of ACN/IPA/water (3 : 3 : 2, v/v/v). After 5 min of vortexing, the mixture was sonicated (WUC-D10H, Daihan Scientific Co., Ltd., Wonju, Korea) for 2 h in an ice bath. After sonication, the mixture was centrifuged at 2682 × *g* for 10 min, and the supernatant was filtered through a 0.22 μm nylon syringe filter (Whatman, Maidstone, UK). Subsequently, 990 μL of the supernatant was transferred to a new centrifuge tube, and 10 μL of the internal standard solution (L-[^13^C_1_] phenylalanine, 0.3 mg mL^−1^) was added before analysis. All samples were prepared in quadruplicate. To monitor the analytical stability and reproducibility of the LC-QTOF/MS system, a pooled quality control (QC) sample was prepared by combining equal volumes of each analytical sample. The QC samples were analyzed in quadruplicate and injected after every four analytical samples throughout the batch run. This strategy ensured consistent system performance and data reliability during acquisition.

#### 2.3.2 LC-Q-TOF/MS

Liquid chromatography coupled with quadrupole time-of-flight mass spectrometry (LC-Q-TOF/MS) system comprised an Agilent 1260 Infinity II series (Agilent Technologies, Santa Clara, CA, USA) coupled to an Agilent 6530 Q-TOF/MS (Agilent Technologies) equipped with an electrospray ionization (ESI) source. The analytical column was a YMC-Pack Pro C18 (150 × 4.6 mm, 3 μm) column (YMC Co., Kyoto, Japan). The mobile phases were 0.1% formic acid in ACN (A) and 0.2% formic acid in water (B) with the programmed gradient elution as follows: 0–30 min, 85% B; 30–35 min, 0% B; 35–35.1 min, 85% B; 35.1–45 min, 85% B, at a flow rate of 0.6 mL min^−1^. The temperature of the column oven was maintained at 30 °C, and the injection volume was 5 μL. MS analysis was acquired in both positive and negative modes, and auto-MS/MS scans were used at a mass-to-charge ratio (*m*/*z*) of 50–1000 *m*/*z*. The MS parameters were set as follows: collision energy, 0, 10, and 20 V; gas temperature, 280 °C; drying gas, 12 L min^−1^; nebulizer, 35 psi; sheath gas temperature, 350 °C; sheath gas flow, 11 L min^−1^; capillary voltage, 4000 V; fragmentor, 100 V; and octopole RF Vpp, 750 V.

### 2.4 Multivariate data processing and statistical analysis

Data acquisition and processing were performed using Agilent MassHunter Qualitative Analysis software (Version 10.0, Agilent Technologies). MS spectra from the LC-Q-TOF/MS analysis were analyzed using the METLIN database B 08.00. The quantification of each metabolite was based on the relative metabolite abundance, which was calculated by comparing the chromatographic peak areas of each sample with the peak areas of the internal standards added to the metabolite extract.

The SIMCA-P software package (Umetrics, Umea, Sweden) was used to perform principal component analysis (PCA), partial least-squares discriminant analysis (PLS-DA) and orthogonal partial least-squares discriminant analysis (OPLS-DA). Unsupervised data analysis, including PCA, was used as exploratory data analysis to show the analytical relationships between samples. OPLS-DA was applied to simulate samples in the retention of established classes *Y*. In the OPLS-DA output, the variable importance in projection (VIP) score was used as a crucial screening parameter for identifying metabolites that exhibited significant differences between AR and ARE samples. The OPLS-DA models were also examined using *R*^2^*Y* and *Q*^2^ values from a random permutation test (*n* = 100) in SIMCA-P. The quality of the PLS model was assessed using the cross-validation parameters *R*^2^ and *Q*^2^, which measured the model's prediction accuracy and explained variance, respectively.

## 3 Results and discussion

### 3.1 Identification of functional metabolites in *A. triphylla* root (AR) and *A. triphylla* root hydrothermal extract (ARE)

**Fig. 1:**
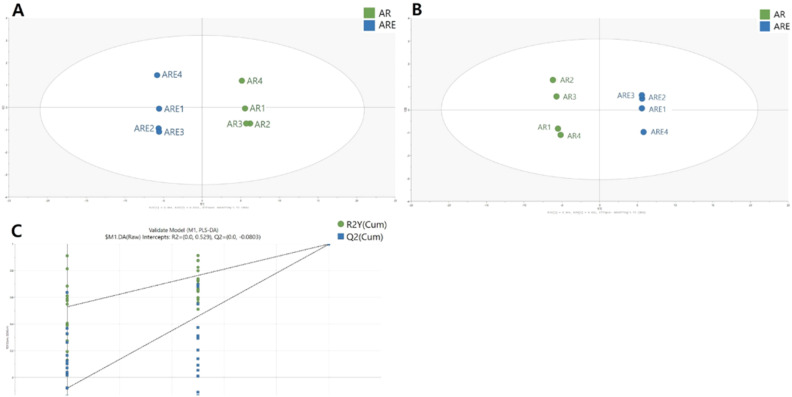
Number of tentatively identified compounds in *A. triphylla* root (AR) and *A. triphylla* root hydrothermal extract (ARE).

LC-Q-TOF/MS was used to analyze phytochemicals in AR and its extract. As a result of the analysis, 38 compounds, including amino acids, phenolic acids, phenolics, carbohydrates, lipids, and miscellaneous compounds (vitamins, terpenes, polyacetylenes, *etc.*), were tentatively identified from AR and ARE in both ESI+ and ESI− modes (Fig. 1). A total of 37 and 28 compounds were detected in AR and ARE, respectively. Previous studies have indicated that *A. triphylla* contains various phytochemicals such as β-sitosterol, lupenone, daucosterol, triphyllol, and adenophoric acid methyl ester.^17^ Details of the 37 compounds identified in AR are summarized in Table S1.† AR included 10 phenolic compounds ((+)-pinoresinol di-*O*-β-d-glucopyranoside, catechin, chlorogenic acid, gallic acid, glehlinoside C, kaempferol, kaempferol-3-*O*-glucoside, lappaol B, tangshenoside I, and vanillic acid-4-β-d-glucoside), 8 organic acids and derivatives (4-*O*-β-d-glucopyranosyl-transcinnamic acid, citric acid, dibutyl sebacate, ferulic acid, quinic acid, sanleng acid, sinapyl alcohol, and sinapic acid), 5 lipids (2-monolinolein, coronaric acid, erucamide, monopalmitin, and stearidonic acid), 2 amino acids (arginine and tryptophan), 4 carbohydrates (glucosyringic acid, sinapoylhexoside, sucrose, and 3-β-d-glucopyranosyloxy-2-butanol), 3 heterocyclic compounds (pyrophaeophorbide A, *O*-isovalery columbianetin, and radicamine A), and 5 miscellaneous, such as vitamins, terpenes, nucleotides and polyacetylene. A total of 28 compounds were detected in ARE, including 8 phenolic compounds, 6 organic acids and derivatives, 5 carbohydrates, 1 lipid, 2 amino acids, 2 heterocyclic compounds, and 4 miscellaneous compounds (Table S2†). Overall, more compounds were detected in AR than in ARE, but there was no significant change between the two samples. More phenolic compounds (10 compounds), organic acids and derivatives (8 compounds), and 5 lipids were detected in AR than in ARE (8 phenolic compounds, 6 organic acids and derivatives, 1 lipid). In particular, 5 lipid compounds were putatively identified in AR, whereas only erucamide was identified in ARE. The reduced number of lipid compounds in ARE may be attributed to their thermal instability and limited extractability under hydrothermal conditions. Due to their strong hydrophobicity, lipids exhibit low solubility in aqueous extraction systems and are prone to structural degradation, such as oxidation or hydrolysis, upon exposure to heat.^34,35^ These properties likely contributed to the reduced lipid extractability under the hydrothermal conditions used in this study. Syringin was also detected only in ARE. Interestingly, the number of phenolic compounds was similar in AR (10) and ARE (8), indicating that they remained unaffected by hydrothermal extraction. Hydrothermal extraction is a commonly used method to extract bioactive compounds from plants.^36,37^ This process is also commonly used in the functional food industry to extract bioactive compounds from various materials.^24,25^ This extract solution can be converted into a powder through spray drying to enhance its shelf life and facilitate its use in the application of health foods.^26^ In previous studies, hydrothermal extraction has been reported to be effective for extracting the useful ingredients of ginseng, ginger, and *Platycodon grandiflorus* root.^28,29,38,39^ Moreover, in traditional East Asian medicine, herbal remedies are commonly prepared through decoction to extract their active constituents.^40,41^ AR contains large amounts of phenolic compounds, among which catechin, kaempferol, vanillic acid-4-β-glucoside, and pinoresinol di-*O*-β-d-glucopyranoside are the major flavonoids. Flavonoids exhibit various biological functions, including anti-inflammatory, antioxidant, antibacterial, and anticancer.^42,43^ Among them, pinoresinol, a lignan compound, is considered a phytoestrogen.^44^ In addition, several metabolites identified in AR and ARE exhibit specific bioactivities, as supported by mechanistic studies. For instance, kaempferol inhibits the NF-κB signaling pathway, thereby regulating inflammatory responses.^45^ Moreover, (+)-pinoresinol di-*O*-β-d-glucopyranoside has demonstrated α-glucosidase inhibitory activity and hepatoprotective effects,^46^ and chlorogenic acid exerts antioxidant and anti-inflammatory properties, contributing to metabolic health.^47^ Ferulic acid and sinapic acid are potent antioxidants that also modulate inflammatory signalling.^48,49^ These bioactive compounds can be used as functional food ingredients to improve consumer health.

### 3.2 Multivariate statistical analysis of the metabolite composition of *A. triphylla* root (AR) and *A. triphylla* root hydrothermal extract (ARE)

**Fig. 2:**
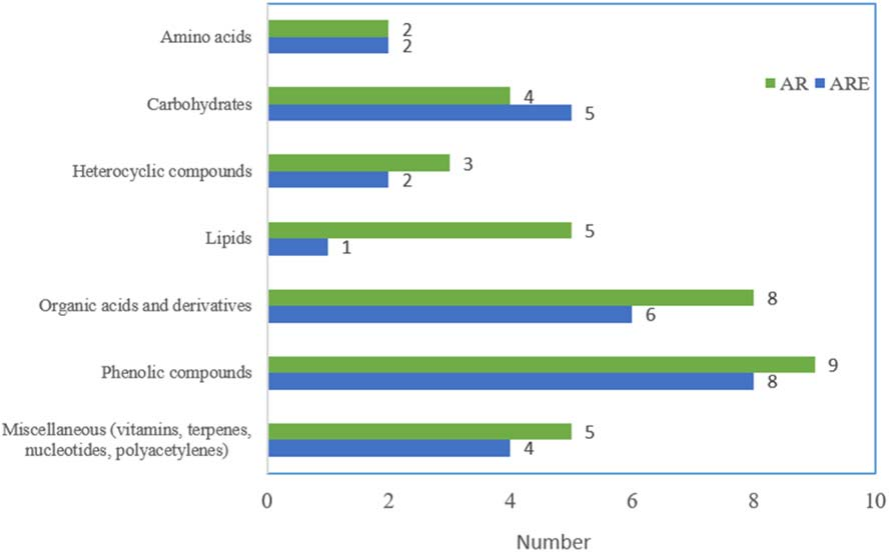
PCA-X score plot (A), PLS-DA score plot (B) and permutation test (C) of *A. triphylla* root (AR) and *A. triphylla* root hydrothermal extract (ARE).

Multivariate statistical analysis is an effective approach for reducing multidimensional datasets and discovering significant patterns of variability and differences between groups in the data.^50^ In this study, multivariate statistical analysis was used to examine metabolite differences between AR and ARE. PCA is an unsupervised learning method extensively used in multivariate statistical analysis, and it can evaluate the clusters and trends of data without providing prior information about the sample class.^51^ In this study, PCA was used to assess the clustering of AR and ARE metabolites. According to the PCA score plot (Fig. 2A), the first principal component (PC1) and the second principal component (PC2) of PCA explained 94.4% and 2.15% of the total variance, respectively, and the AR and ARE groups were separated along PC1. To evaluate the analytical reproducibility and stability of the LC-Q-TOF/MS platform, pooled QC samples were injected at regular intervals throughout the analytical sequence. In the PCA model containing the QC samples (Fig. S1†), the QC samples formed a distinct and tight cluster, separated from the AR and ARE sample groups. Such clustering reflects high signal consistency, instrumental stability, and reliable data acquisition. The model showed strong explanatory and predictive power, with *R*^2^*X* = 0.876 and *Q*^2^ = 0.788, supporting the robustness and reproducibility of the metabolomics workflow. Since PLS-DA has a stronger ability to extract variation information between groups than PCA,^52^ a PLS-DA model was constructed to observe the differences between the AR and ARE groups. As a result, AR and ARE were separated based on PC1 in the PLS-DA model (Fig. 2B). In addition, the *R*^2^*Y* values, which are parameters representing the goodness of fit of the model, and the *Q*^2^ values, which represent the goodness of prediction, were 0.999 and 0.998, respectively, which were higher than 0.5 and close to 1.0, indicating the high correlation and predictability of the model for identifying metabolites between the AR and ARE groups. In addition, permutation tests (100 times) were performed to identify the absence of overfitting in the proposed PLS-DA model. As shown in Fig. 2C, the intercepts of *R*^2^*Y* and *Q*^2^*Y* were 0.529 and −0.0803, respectively, which were higher in the permutation test than in the actual model, indicating excellent predictability and goodness of fit.

**Fig. 3:**
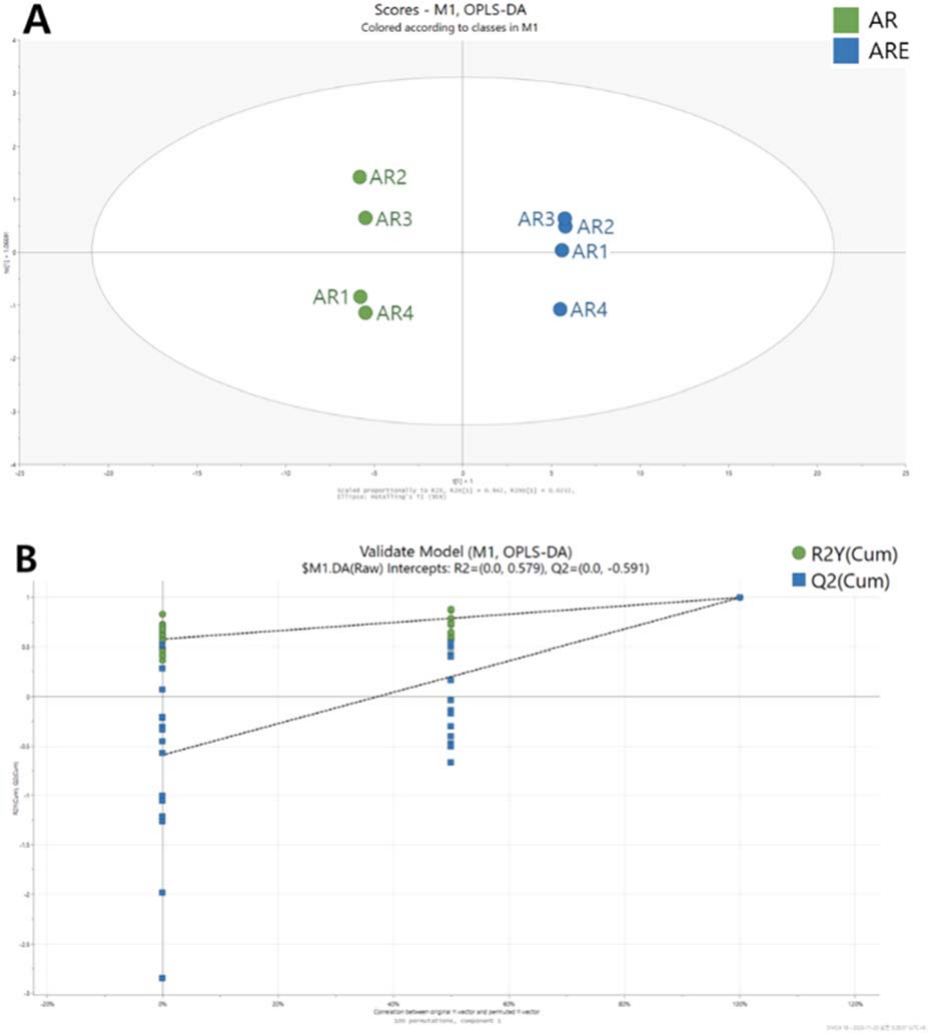
OPLS-DA score plot (A) and permutation test (B) of *A. triphylla* root (AR) and *A. triphylla* root hydrothermal extract (ARE).

**Fig. 4:**
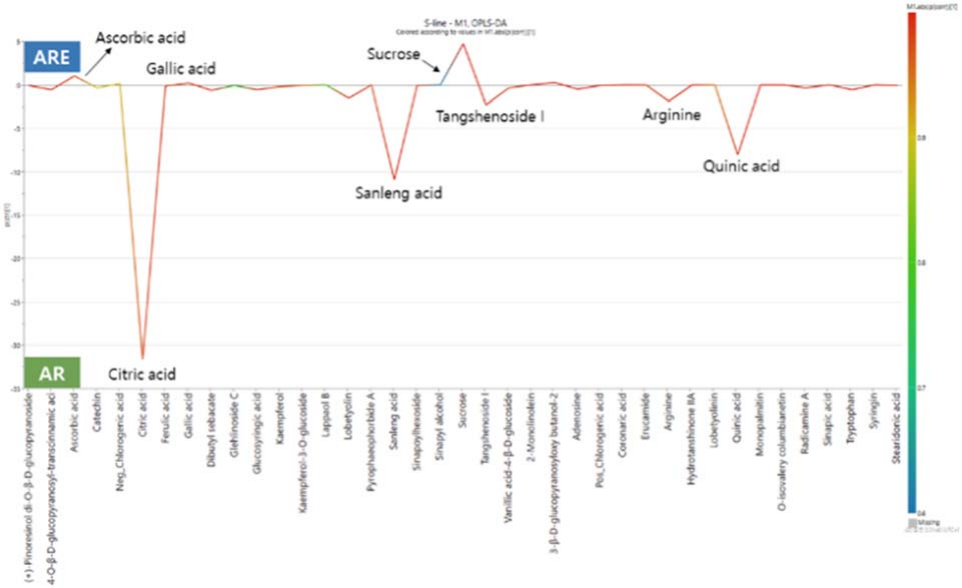
S-line plot of the OPLS-DA of *A. triphylla* root (AR) and *A. triphylla* root hydrothermal extract (ARE).

OPLS-DA is a method that has the same predictive power as PLS-DA but provides a better interpretation of variables than PLS-DA.^53^ The OPLS-DA model in this study separated the AR and ARE groups, similar to PLS-DA (Fig. 3A). In addition, the *R*^2^*Y* and *Q*^2^ parameters of the OPLS-DA model were 0.999 and 0.998, respectively, indicating high correlation and predictability. To confirm the overfitting of the model, a permutation test (100 times) was performed on the OPLS-DA model (Fig. 3B). In the permutation test, the *R*^2^*Y* and *Q*^2^*Y* intercepts were 0.579 and −0.591, respectively, indicating that the OPLS-DA model showed great predictability and was stable and reproducible. In this study, metabolites selected with a VIP score higher than 1 and *p*-value <0.05 in the OPLS-DA model were selected to identify differential metabolites (Table 1). Among the 32 differential metabolites, 21 were identified in both AR and ARE, while 11 were unique to AR. Overall, more types of differential metabolites were identified in AR, although the majority were detected in both AR and ARE. Among these differential metabolites, the largest number of compounds were identified as organic acids and derivatives, followed by phenolic compounds, carbohydrates and lipids. These metabolites improved the discrimination between AR and ARE. The differential metabolite results are visualized in the S-line plot. The OPLS-DA S-line plot visualizes the covariance and correlation coefficient between variables obtained from the OPLS-DA model and can be used to determine if an increase or decrease in a variable is correlated with the classification score.^54^ The *y*-axis on the right represents the correlation coefficient between the variable and the classification score and shows the strength of the correlation linearly, with a redder color indicating a higher absolute value of the correlation coefficient. As shown in Fig. 4, eight compounds, namely ascorbic acid, citric acid, gallic acid, sanleng acid, sucrose, tangshenoside I, arginine, and quinic acid, were identified as the main differential metabolites of AR and ARE. The relative contents of these eight differential metabolites are shown in Fig. S2.† These metabolites are indicated on representative chromatograms (Fig. S3†), and their chemical structures are presented in Fig. S4.† Citric acid was higher in AR than in ARE, followed by sanleng acid and quinic acid in AR. On the other hand, ascorbic acid, gallic acid, and sucrose had relatively high ARE contents.

**Table 1:** Variable importance in the projection (VIP) scores of OPLS-DA

No.	Compound name	Mode	VIP score	CV
1	Tangshenoside I	(−)	1.026	0.036
2	3-β-d-Glucopyranosyloxy-2-butanol	(+)	1.026	0.058
3	Tryptophan	(+)	1.026	0.052
4	Hydrotanshinone IIA	(+)	1.025	0.060
5	Coronaric acid	(+)	1.025	0.036
6	Lobetyolin	(−)	1.025	0.047
7	Sucrose	(−)	1.025	0.099
8	Monopalmitin	(+)	1.025	0.045
9	Stearidonic acid	(+)	1.025	0.057
10	Chlorogenic acid	(+)	1.024	0.055
11	Sanleng acid	(−)	1.024	0.049
12	2-Monolinolein	(+)	1.024	0.107
13	Dibutyl sebacate	(−)	1.024	0.077
14	Adenosine	(+)	1.024	0.055
15	Radicamine A	(+)	1.023	0.080
16	Glucosyringic acid	(−)	1.023	0.088
17	Arginine	(+)	1.022	0.070
18	*O*-Isovalery columbianetin	(+)	1.022	0.071
19	Syringin	(+)	1.021	0.109
20	4-*O*-β-d-Glucopyranosyl-transcinnamic acid	(−)	1.019	0.092
21	Sinapic acid	(+)	1.018	0.110
22	(+)-Pinoresinol di-*O*-β-d-glucopyranoside	(−)	1.018	0.182
23	Gallic acid	(−)	1.018	0.092
24	Quinic acid	(−)	1.017	0.122
25	Ascorbic acid	(−)	1.017	0.141
26	Sinapoylhexoside	(−)	1.015	0.145
27	Ferulic acid	(−)	1.013	0.169
28	Pyrophaeophorbide A	(−)	1.009	0.218
29	Citric acid	(−)	1.009	0.251
30	Kaempferol	(−)	1.008	0.200
31	Erucamide	(+)	1.006	0.235
32	Vanillic acid-4-β-d-glucoside	(−)	1.004	0.213

Water is a non-toxic, readily available, and environmentally friendly solvent that primarily extracts polar components from plant matrices, but increasing the extraction temperature can degrade the cell walls, releasing water-soluble substances with various polarities.^55^ The hydrothermal extraction condition at 95 °C used in this study may have facilitated the release of water-soluble compounds, which is reflected in the relatively higher levels of polar metabolites such as sucrose, gallic acid, and ascorbic acid in ARE compared to AR. Similar trends have been reported in previous studies. Ferreira *et al.* (2019) demonstrated that the extraction of phenolic compounds, including phlorotannins, from *Fucus vesiculosus* increased with temperature during hydrothermal extraction, with phlorotannins being effectively and stably extracted even near 100 °C.^56^ Furthermore, studies on other medicinal plants such as lemon balm,^57^ thyme,^58^ and *Bidens pilosa*^59^ have reported that polyphenols and flavonoids can be efficiently extracted under hydrothermal or subcritical water conditions at or near 100 °C.

Moreover, phenolic compounds such as gallic acid are easily extracted as the polyphenol structure is transformed at high temperatures.^60^ Ascorbic acid acts as an antioxidant that prevents the oxidation of other components and increases stability,^61^ and these properties may increase the ascorbic acid content after hydrothermal extraction conditions. Therefore, the hydrothermal extraction method used in the preparation of ARE may have facilitated the release of certain water-soluble bioactive metabolites more effectively than AR. This observation is consistent with the results of the differential metabolite analysis. According to the analysis, most major metabolites were commonly detected in both AR and ARE, whereas tangshenoside I was identified exclusively in AR. This compound, a syringin derivative primarily found in *Codonopsis lanceolata* has potential activity related to muscle atrophy improvement.^62^ Other key metabolites such as arginine, ascorbic acid, citric acid, gallic acid, quinic acid, sucrose, and sanleng acid were detected in both samples. Among them, ascorbic acid, gallic acid, and sucrose were more abundant in ARE, whereas arginine, citric acid, quinic acid, and sanleng acid were present at higher levels in AR. These metabolites are associated with various physiological activities. For instance, gallic acid reduces oxidative stress and exerts anti-inflammatory effects through modulation of the MAPK and NF-κB pathways.^63^ Ascorbic acid acts as an antioxidant that enhances the stability of other compounds. Citric acid plays a crucial role in cellular energy metabolism and immune regulation,^64^ whereas quinic acid has been linked to anti-inflammatory, antidiabetic, and analgesic effects.^65^ Sanleng acid is a type of organic acid found in *Sparganii rhizoma*, but its specific physiological activity mechanism has not yet been reported.^66^ Overall, these results indicate that hydrothermal extraction can influence the composition of bioactive compounds, highlighting the potential of these extracts as functional food ingredients. However, further study is necessary to confirm the bioactive effects of the treatment.

## 4 Conclusion

This study examined the metabolomic alterations observed in dried AR and its corresponding hot-water extracts using LC-Q-TOF/MS. A total of 37 and 28 compounds were identified in AR and ARE, respectively. Untargeted LC-Q-TOF/MS-based metabolomics provided comprehensive metabolite profiles of AR and its extract. These findings provide basic information for the product development of AR. Further research is ongoing to enhance the quality of AR in the development of functional foods. However, the biological significance of the identified metabolite changes requires further validation. Future studies will focus on the targeted quantification and biological evaluation of key AR metabolites to better understand their roles in physiological functions and therapeutic potential.

## Abbreviations

LC-Q-TOF/MS: Liquid chromatography-quadrupole-time-of-flight mass spectrometry
AR: *A. triphylla* root
ARE: *A. triphylla* root hydrothermal extract
PCA: Principal component analysis
PLS-DA: Partial least-squares discriminant analysis
OPLS-DA: Orthogonal partial least-squares discriminant analysis
VIP: Variable importance in projection

## Data availability

The data supporting this article have been included as part of the ESI.†

## Author contributions

Investigation, formal analysis, and writing – original draft, Y. K. (Yoonjeong Kim); investigation and formal analysis, J. P.; formal analysis, Y. Y.; conceptualization, C. J.; conceptualization, D. L.; conceptualization, I. L.; writing – review and editing, Y. K. (Younghwa Kim). All the authors have read and agreed to the published version of the manuscript.

## Conflicts of interest

There are no conflicts to declare.

## Supplementary Material

RA-015-D5RA00186B-s001

## References

[cit1] Wang J. H., Byun J., Pennathur S. (2010). Semin. Nephrol..

[cit2] Zeki O. C., Eylem C. C., Recber T., Kir S., Nemutlu E. (2020). J. Pharm. Biomed. Anal..

[cit3] Fu J., Zhang L. L., Li W., Zhang Y., Zhang Y., Liu F., Zou L. (2022). Food Chem..

[cit4] Lacalle-Bergeron L., Izquierdo-Sandoval D., Sancho J. V., López F. J., Hernández F., Portolés T. (2021). Trac. Trends Anal. Chem..

[cit5] Yuliana N. D., Hunaefi D., Goto M., Ishikawa Y. T., Verpoorte R. (2022). Crit. Rev. Food Sci. Nutr..

[cit6] Li R., Sun Z., Zhao Y., Li L., Yang X., Cen J., Chen S., Li C., Wang Y. (2021). Food Chem..

[cit7] Xiao Y., Liu H., Du P., Liang X., Li H., Lu Q., Li S., Liu H., Hong Y., Varshney R. K., Chen X. (2022). Food Chem..

[cit8] Utpott M., Rodrigues E., Rios A. O., Mercali G. D., Flores S. H. (2022). Food Chem..

[cit9] Martinez-Moreno A., Perez-Porras P., Bautista-Ortin A. B., Gomez-Plaza E., Vallejo F. (2024). Foods.

[cit10] Gao Z., Zhou M. C., Lin J., Lu Y., Liu S. Q. (2024). Food Chem.:X.

[cit11] Johanningsmeier S. D., Harris G. K., Klevorn C. M. (2016). Annu. Rev. Food Sci. Technol..

[cit12] Lei H., Zhang Y., Ye J., Cheng T., Liang Y., Zu X., Zhang W. (2021). J. Ethnopharmacol..

[cit13] Guo S., Qiu S., Cai Y., Wang Z., Yang Q., Tang S., Xie Y., Zhang A. (2023). Front. Chem..

[cit14] Gu X., Gao X., Cheng J., Xia C., Xu Y., Yang L., Zhou M. (2021). Biomed. Pharmacother..

[cit15] Commisso M., Strazzer P., Toffali K., Stocchero M., Guzzo F. (2013). Comput. Struct. Biotechnol. J..

[cit16] Zhou L., Xu J. D., Zhou S. S., Mao Q., Kong M., Shen H., Li X. Y., Duan S. M., Xu J., Li S. L. (2016). J. Chromatogr. A.

[cit17] Konno C., Saito T., Oshima Y., Hikino H., Kabuto C. (1981). Planta Med..

[cit18] Ham Y. A., Choi H. J., Chung M. J., Ham S. S. (2009). J. Korean Soc. Food Nutr. Sci..

[cit19] Kim J. H., Hong J. Y., Shin S. R., Yoon K. Y. (2009). Int. J. Nutr. Food Sci..

[cit20] Akihisa T., Yasukawa K., Oinuma H., Kasahara Y., Yamanouchi S., Takido M., Kumaki K., Tamura T. (1996). Phytochemistry.

[cit21] Lee Y. M., Bae J. H., Nam S. Y., Jang H. H., Park M. Y., Kim J. B., Park D. S., Kim H. R. (2013). Food Sci. Biotechnol..

[cit22] Devkota H. P., Hori K. (2020). Nepal J. Biotechnol..

[cit23] Chun J., Kang M., Kim Y. S. (2014). Tumor Biol..

[cit24] Munir M., Kheirkhah H., Baroutian S., Quek S. Y., Young B. R. (2018). J. Clean. Prod..

[cit25] Maqbool Z., Khalid W., Atiq H. T., Koraqi H., Javaid Z., Alhag S. K., Al-Shuraym L. A., Bader D. M. D., Almarzuq M., Afifi M., Al-Farga A. (2023). Molecules.

[cit26] Asano N., Nishida M., Miyauchi M., Ikeda K., Yamamoto M., Kizu H., Kameda Y., Watson A. A., Nash R. J., Fleet G. W. (2000). Phytochemistry.

[cit27] Yoon S. Y., Kim S. W., Hyun T. K. (2024). Heliyon.

[cit28] Yulianto M. E., Paramita V., Amalia R., Wahyuningsih N., Nyamiati R. D. (2022). Mater. Today: Proc..

[cit29] Zhou Y., Jin T., Gao M., Luo Z., Mutahir S., Shi C., Xie T., Lin L., Xu J., Liao Y., Chen M., Deng H., Zheng M., Shan J. (2023). Chin. Med..

[cit30] Yoon Y. P., Lee H. J., Lee D. U., Lee S. K., Hong J. H., Lee C. J. (2015). Tuberc. Respir. Dis..

[cit31] Park W. S., Kim H. J., Choe S. J., Khalil A. A. K., Akter K. M., Shin M. C., Chung H. J., Park J. H., Ahn M. J. (2020). Rev. Bras. Farmacogn..

[cit32] Vu T. P. D., Kim K. T., Pham Y., Bao H., Kang J. S. (2017). Anal. Sci. Technol..

[cit33] Yoon S. Y., Kim K. H., Hyun T. K. (2023). J. Plant Biotechnol..

[cit34] Zhang H., Xu Y., Zhao C., Xue Y., Tan D., Wang S., Jia M., Wu H., Ma A., Chen G. (2022). Food Res. Int..

[cit35] Hu Q., Zhang J., He L., Wei L., Xing R., Yu N., Huang W., Chen Y. (2024). Food Res. Int..

[cit36] Kim I. S., Yang M. R., Lee O. H., Kang S. N. (2011). Int. J. Mol. Sci..

[cit37] Chou S. T., Lin T. H., Peng H. Y., Chao W. W. (2019). Life Sci..

[cit38] Krishnaiah D., Nithyanandam R., Sarbatly R. (2014). Crit. Rev. Food Sci. Nutr..

[cit39] Li X., Han J. S., Park Y. J., Kang S. J., Kim J. S., Nam K. Y., Lee K. T., Choi J. E. (2009). Korean J. Crop Sci..

[cit40] Li F. S., Weng J. K. (2017). Nat. Plants.

[cit41] Gao Y., Dong Y., Guo Q., Wang H., Feng M., Yan Z., Bai D. (2022). Molecules.

[cit42] Juca M. M., Cysne Filho F. M. S., de Almeida J. C., Mesquita D. D. S., Barriga J. R. M., Dias K. C. F., Barbosa T. M., Vasconcelos L. C., Leal L., Ribeiro J. E., Vasconcelos S. M. M. (2020). Nat. Prod. Res..

[cit43] Kumar S., Pandey A. K. (2013). Sci. World J..

[cit44] López-Biedma A., Sánchez-Quesada C., Beltrán G., Delgado-Rodríguez M., Gaforio J. J. (2016). BMC Complementary Altern. Med..

[cit45] Hosseini A., Alipour A., Baradaran Rahimi V., Askari V. R. (2023). Biofactors.

[cit46] Youssef F. S., Ashour M. L., El-Beshbishy H. A., Ahmed Hamza A., Singab A. N. B., Wink M. (2020). J. Pharm. Pharmacol..

[cit47] Huang J., Xie M., He L., Song X., Cao T. (2023). Front. Pharmacol..

[cit48] Zduńska K., Dana A., Kolodziejczak A., Rotsztejn H. (2018). Skin Pharmacol. Physiol..

[cit49] Pandi A., Kalappan V. M. (2021). Mol. Biol. Rep..

[cit50] Sussulini A. (2017). Adv. Exp. Med. Biol..

[cit51] Jolliffe I. T., Cadima J. (2016). Philos. Trans. A Math. Phys. Eng. Sci..

[cit52] Boccard J., Veuthey J. L., Rudaz S. (2010). J. Sep. Sci..

[cit53] Nitta K., Lavina W. A., Pontrelli S., Liao J. C., Putri S. P., Fukusaki E. (2017). J. Biosci. Bioeng..

[cit54] Bervoets L., Massa G., Guedens W., Louis E., Noben J. P., Adriaensens P. (2017). Diabetol. Metab. Syndr..

[cit55] Gallina L., Cravotto C., Capaldi G., Grillo G., Cravotto G. (2022). Processes.

[cit56] Ferreira R. M., Ramalho Ribeiro A., Patinha C., Silva A. M., Cardoso S. M., Costa R. (2019). Molecules.

[cit57] Atanasova A., Petrova A., Teneva D., Ognyanov M., Georgiev Y., Nenov N., Denev P. (2023). Antioxidants.

[cit58] Vergara-Salinas J. R., Pérez-Jiménez J., Torres J. L., Agosin E., Pérez-Correa J. R. (2012). J. Agric. Food Chem..

[cit59] Gbashi S., Njobeh P., Steenkamp P., Madala N. (2017). CyTA--J. Food.

[cit60] Nirmal N. P., Khanashyam A. C., Mundanat A. S., Shah K., Babu K. S., Thorakkattu P., Al-Asmari F., Pandiselvam R. (2023). Foods.

[cit61] Njus D., Kelley P. M., Tu Y. J., Schlegel H. B. (2020). Free Radic. Biol. Med..

[cit62] Kim T. Y., Park K. T., Choung S. Y. (2022). Phytomedicine.

[cit63] Chu C., Ru H., Chen Y., Xu J., Wang C., Jin Y. (2024). Acta Biochim. Biophys. Sin..

[cit64] Zotta A., Zaslona Z., O’Neill L. A. (2020). J. Cell. Signal..

[cit65] Benali T., Bakrim S., Ghchime R., Benkhaira N., El Omari N., Balahbib A., Taha D., Zengin G., Hasan M. M., Bibi S., Bouyahya A. (2024). Biotechnol. Genet. Eng. Rev..

[cit66] Lu X., Zheng Y., Wen F., Huang W., Chen X., Ruan S., Gu S., Hu Y., Teng Y., Shu P. (2021). Sci. Rep..

