# Supplementary material for: Profiling of phytochemicals in Adenophora triphylla using LC-Q-TOF/MS-based untargeted metabolomics
Source: RSC Adv. 2025 Jun 2;15(23):18275–82. doi: 10.1039/d5ra00186b (PMC12127841; doi:10.1039/d5ra00186b)
Supplement: RA-015-D5RA00186B-s001 [file RA-015-D5RA00186B-s001.pdf]

Supplementary Information (SI) for RSC Advances.
This journal is © The Royal Society of Chemistry 2025

Supplementary data

Profiling of phytochemicals in *Adenophora triphylla* using LC-Q-TOF/MS based untargeted metabolomics

Yoonjeong Kim, [a] Jiye Pyeon, [a] Yeong Eun Yu, [b] Cheol-jong Jung, [b] Do Sang Lee, [c] Im-Joung La [c] and Younghwa Kim[*a]

[a.] *Department of Food Science and Biotechnology, Kyungsung University, Busan, 48434, Republic of Korea*

[b.] *Central Research Center, Okchundang Inc., Daegu 41059, Republic of Kore*

[c.] *Atomy R&D Center, Gongju 32511, Republic of Korea*

[*]Corresponding author. Department of Food Science and Biotechnology, Kyungsung University, Busan, 48434, Republic of Korea. Tel: +82-51-663-4652. Fax: +82-51-622-4986

E-mail address: younghwakim@ks.ac.kr (Y. Kim).

Table S1. Identification of the chemical compounds from *A. triphylla* root (AR)

Ion mode	No.	Compound	Molecular formula	Retention time (min)	Calculated m/z	Experimental m/z	Fragment ions (m/z)	Adducts	Mass error (ppm)
Negative	1	(+)-Pinoresinol di-*O*-*β*-D-glucopyranoside	$C_{32}H_{42}O_{16}$	9.884	681.24	681.2458	339.12	[M-H]-	8.514
	2	4-*O*-*β*-D-glucopyranosyl-transcinnamic acid	$C_{15}H_{18}O_8$	8.816	371.0984	371.0988	117.0187	[M+HCOO]-	1.078
	3	Ascorbic acid	$C_6H_8O_6$	3.403	175.0248	175.0246	219.1465, 115.0037, 87.0085, 59.0137	[M-H]-	-1.143
	4	Catechin	$C_{15}H_{14}O_6$	2.909	289.0718	289.0703	215.0372, 101.0244, 165.0395	[M-H]-	-5.189
	5	Chlorogenic acid	$C_{16}H_{18}O_9$	2.87	353.0878	353.0873	191.0571	[M-H]-	-1.416
	6	Citric acid	$C_6H_8O_7$	3.306	191.0197	191.0199	173.0092, 111.0084	[M-H]-	1.047
	7	Dibutyl sebacate	$C_{18}H_{34}O_4$	18.209	313.2384	313.2388	199.0919, 155.1076	[M-H]-	1.277
	8	Ferulic acid	$C_{10}H_{10}O_4$	10.631	193.0506	193.0499	179.0561, 147.0272, 135.0423, 134.0308, 101.0245	[M-H]-	-3.626
	9	Gallic acid	$C_7H_6O_5$	3.494	169.0142	169.015	125.0258	[M-H]-	4.733
	10	Glehlinoside C	$C_{26}H_{32}O_{13}$	2.575	551.177	551.1768	345.1364	[M-H]-	-0.363
	11	Glucosyringic acid	$C_{15}H_{20}O_{10}$	4.299	359.0984	359.0969	179.0567	[M-H]-	-4.177
	12	Kaempferol	$C_{15}H_{10}O_6$	22.978	285.0405	285.0415	153.0205, 258.0545	[M-H]-	3.508
	13	Kaempferol-3-*O*-glucoside	$C_{21}H_{20}O_{11}$	9.409	447.0933	447.0955	161.0455, 131.0679	[M-H]-	4.921
	14	Lappaol B	$C_{31}H_{34}O_9$	2.652	595.2185	595.2192	301.1091, 518.1986	[M+HCOO]-	1.176
	15	Lobetyolin	$C_{20}H_{28}O_8$	11.177	441.1766	441.1768	215.1097	[M+HCOO]-	0.453
	16	Pyrophaeophorbide A	$C_{33}H_{34}N_4O_3$	7.805	579.2613	579.2608	493.2274	[M+HCOO]-	-0.863
	17	Quinic acid	$C_7H_{12}O_6$	2.782	191.0561	191.0571	173.0461, 127.0409	[M-H]-	5.234
	18	Sanleng acid	$C_{18}H_{34}O_5$	15.812	329.2333	329.2336	311.2301, 199.1310, 185.1152	[M-H]-	0.911
	19	Sinapoylhexoside	$C_{17}H_{22}O_{10}$	9.694	385.114	385.1147	223.0508, 247.0510	[M-H]-	1.818
	20	Sinapyl alcohol	$C_{11}H_{14}O_4$	11.644	209.0819	209.0792	208.0536, 181.0694	[M-H]-	-12.914
	21	Sucrose	$C_{12}H_{22}O_{11}$	2.614	341.1089	341.1098	71.0135, 89.0241, 101.0242, 179.0563	[M-H]-	2.638
	22	Tangshenoside I	$C_{29}H_{42}O_{18}$	7.484	677.2298	677.2294	497.1655, 453.1713	[M-H]-	-0.591

	23	Vanillic acid-4-*β*-D-glucoside	$C_{14}H_{18}O_9$	3.504	329.0878	329.0878	311.0732, 253.0795, 151.0091	[M-H]-	0.000
Positive	1	2-Monolinolein	$C_{21}H_{38}O_4$	32.563	377.2662	377.2674	263.2382	[M+Na]+	3.181
	2	3-*β*-D-glucopyranosyloxy-2-butanol	$C_{10}H_{20}O_7$	5.705	253.1282	253.1294	222.115	[M+H]+	4.741
	3	Adenosine	$C_{10}H_{13}N_5O_4$	2.376	268.104	268.1039	218.1032, 136.0614, 94.0498, 57.0323	[M+H]+	-0.373
	4	Arginine	$C_6H_{14}N_4O_2$	2.066	175.119	175.1189	60.0538, 70.0651, 72.0804, 114.1007, 116.0705, 130.0975, 158.0919	[M+H]+	-0.571
	5	Chlorogenic acid	$C_{16}H_{18}O_9$	6.522	355.1024	355.1021	163.0481	[M+H]+	-0.845
	6	Coronaric acid	$C_{18}H_{32}O_3$	3.511	319.2244	319.227	95.0845, 147.1160, 277.2173	[M+Na]+	8.145
	7	Erucamide	$C_{22}H_{43}NO$	3.953	338.3417	338.3412	321.3144, 128.1021, 114.0900, 322.3107, 303.3090	[M+H]+	-1.478
	8	Hydrotanshinone IIA	$C_{19}H_{18}O_4$	2.536	311.1278	311.1272	217.1213	[M+H]+	-1.928
	9	Lobetyolinin	$C_{26}H_{38}O_{13}$	9.826	581.2205	581.2201	379.1742	[M+Na]+	-0.688
	10	Monopalmitin	$C_{19}H_{38}O_4$	24.924	353.2662	353.268	239.2311, 173.1300, 137.0956, 107.0830	[M+Na]+	5.095
	11	*O*-Isovalery columbianetin	$C_{19}H_{22}O_5$	9.92	331.154	331.1539	229.0893	[M+H]+	-0.302
	12	Radicamine A	$C_{12}H_{17}NO_5$	2.309	256.1179	256.1182	226.1079	[M+H]+	1.171
	13	Sinapic acid	$C_{11}H_{12}O_5$	13.904	225.0757	225.0759	207.0682, 225.0755	[M+H]+	0.889
	14	Stearidonic acid	$C_{18}H_{28}O_2$	27.38	277.2162	277.2162	217.1924, 203.1784, 201.1606, 105.0691	[M+H]+	0.000
	15	Tryptophan	$C_{11}H_{12}N_2O_2$	4.72	205.0972	205.0972	188.0705, 159.0909, 146.0633, 144.0802, 143.0712, 91.0534	[M+H]+	0.000

Table S2. Identification of the chemical compounds from *A. triphylla* root hydrothermal extract (ARE)

Ion mode	Peak no.	Compound name	Molecular formula	Retention time (min)	Calculated m/z	Experimental m/z	Fragment ions (m/z)	Adducts	Mass error (ppm)
Negative	1	(+)-Pinoresinol di-*O*-*β*-D-glucopyranoside	$C_{32}H_{42}O_{16}$	9.856	681.24	681.2461	501.1761, 339.1273	[M-H]-	8.954
	2	4-*O*-*β*-D-glucopyranosyl-transcinnamic acid	$C_{15}H_{18}O_8$	8.814	371.0984	371.0994	162.0503, 117.0205	[M+HCOO]-	2.695
	3	Ascorbic acid	$C_6H_8O_6$	3.437	175.0248	175.0248	115.0039, 87.0088, 59.0138	[M-H]-	0.000
	4	Catechin	$C_{15}H_{14}O_6$	2.941	289.0718	289.0705	215.0345, 191.0204, 101.024	[M-H]-	-4.497
	5	Chlorogenic acid	$C_{16}H_{18}O_9$	2.879	353.0878	353.087	191.0572	[M-H]-	-2.266
	6	Citric acid	$C_6H_8O_7$	3.286	191.0197	191.02	173.0091, 111.0084	[M-H]-	1.571
	7	Dibutyl sebacate	$C_{18}H_{34}O_4$	18.124	313.2384	313.2386	155.1097	[M-H]-	0.638
	8	Gallic acid	$C_7H_6O_5$	3.512	169.0142	169.0147	125.0247	[M-H]-	2.958
	9	Glehlinoside C	$C_{26}H_{32}O_{13}$	2.543	551.177	551.1762	345.1312	[M-H]-	-1.451
	10	Glucosyringic acid	$C_{15}H_{20}O_{10}$	4.267	359.0984	359.096	179.0566, 137.0633	[M-H]-	-6.683
	11	Kaempferol-3-*O*-glucoside	$C_{21}H_{20}O_{11}$	9.362	447.0933	447.0939	161.0459, 131.0701	[M-H]-	1.342
	12	Lappaol B	$C_{31}H_{34}O_9$	2.629	595.2185	595.2181	518.1942, 301.1120	[M+HCOO]-	-0.672
	13	Lobetyolin	$C_{20}H_{28}O_8$	11.212	441.1766	441.1787	215.1031	[M+HCOO]-	4.760
	14	Quinic acid	$C_7H_{12}O_6$	2.801	191.0561	191.0571	173.0452, 127.0415	[M-H]-	5.234
	15	Sanleng acid	$C_{18}H_{34}O_5$	15.901	329.2333	329.2372	185.1158, 311.2205	[M-H]-	11.846
	16	Sinapoylhexoside	$C_{17}H_{22}O_{10}$	9.525	385.114	385.1159	205.0493, 223.0545, 247.0501	[M-H]-	4.934
	17	Sinapyl alcohol	$C_{11}H_{14}O_4$	11.655	209.0819	209.0793	208.0548, 181.0679	[M-H]-	-12.435
	18	Sucrose	$C_{12}H_{22}O_{11}$	2.609	341.1089	341.1109	71.0134, 89.0240, 179.0567, 101.0239	[M-H]-	5.863
	19	Vanillic acid-4-*β*-D-glucoside	$C_{14}H_{18}O_9$	3.474	329.0878	329.0869	311.0756, 269.0643, 151.0067	[M-H]-	-2.735
Positive	1	3-*β*-D-glucopyranosyloxy-2-butanol	$C_{10}H_{20}O_7$	2.995	253.1282	253.1297	222.1142	[M+H]+	5.926
	2	Adenosine	$C_{10}H_{13}N_5O_4$	2.379	268.104	268.1041	218.1046, 136.0626, 57.0337	[M+H]+	0.373
	3	Arginine	$C_6H_{14}N_4O_2$	2.049	175.119	175.119	158.0922, 60.0541, 70.0652, 72.0803, 114.1032, 116.0707, 130.0976	[M+H]+	0.373
	4	Erucamide	$C_{22}H_{43}NO$	4.033	338.3417	338.3418	322.3151, 321.3147, 128.1033,	[M+H]+	0.296

						114.0910		
5	Lobetyolinin	$C_{26}H_{38}O_{13}$	9.829	581.2205	581.2208	379.1793	[M+Na]+	0.516
6	*O*-Isovalery columbianetin	$C_{19}H_{22}O_5$	9.967	331.154	331.1536	229.0837	[M+H]+	-1.208
7	Radicamine A	$C_{12}H_{17}NO_5$	2.243	256.1179	256.1188	226.1057	[M+H]+	3.514
8	Syringin	$C_{17}H_{24}O_9$	5.742	395.1313	395.1315	377.1296, 299.0977, 245.0841, 233.0805	[M+Na]+	0.506
9	Tryptophan	$C_{11}H_{12}N_2O_2$	4.701	205.0972	205.0966	188.0674, 146.0588, 143.0703, 159.0953	[M+H]+	-2.925

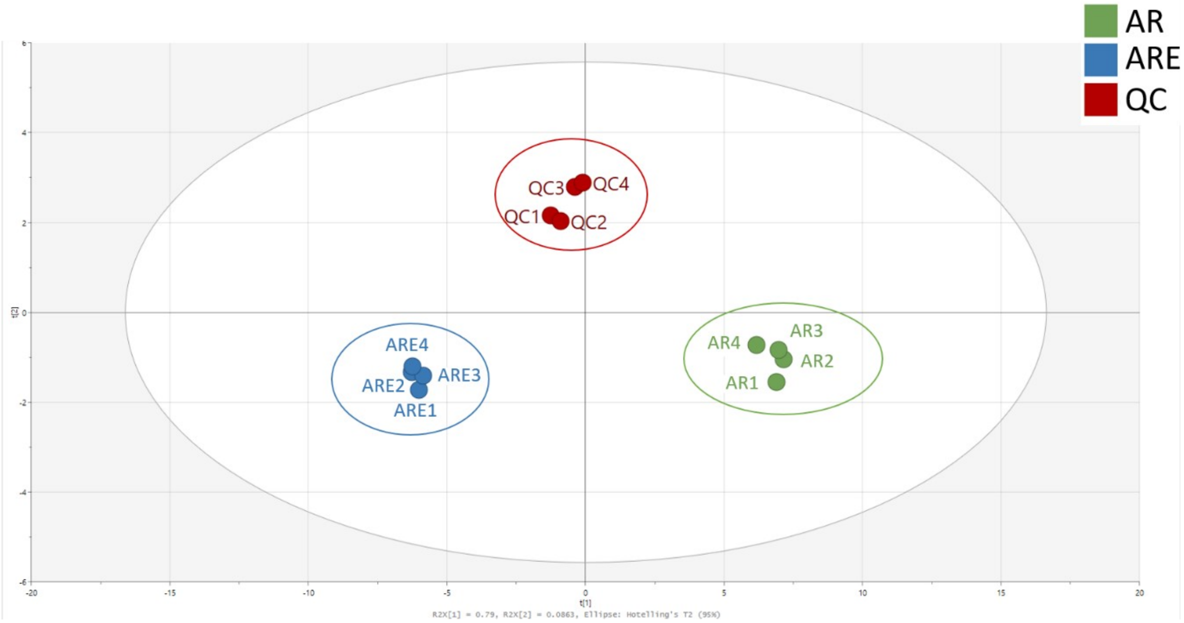

Fig. S1. Principal component analysis (PCA) score plot including quality control (QC) samples. Green, blue and red circles represent AR, ARE and QC samples, respectively.

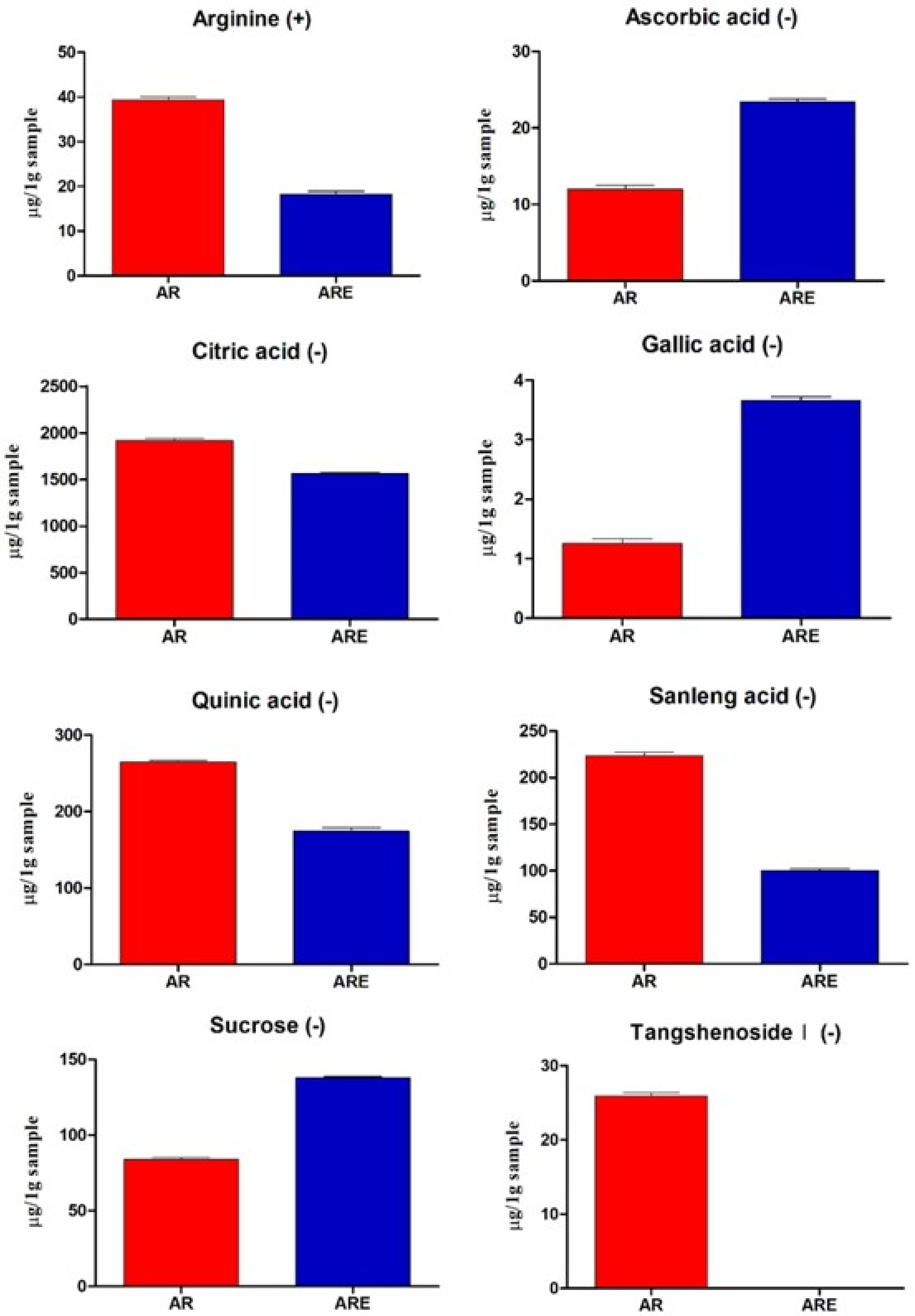

Fig. S2. The relative contents of differential metabolites between AR and ARE.

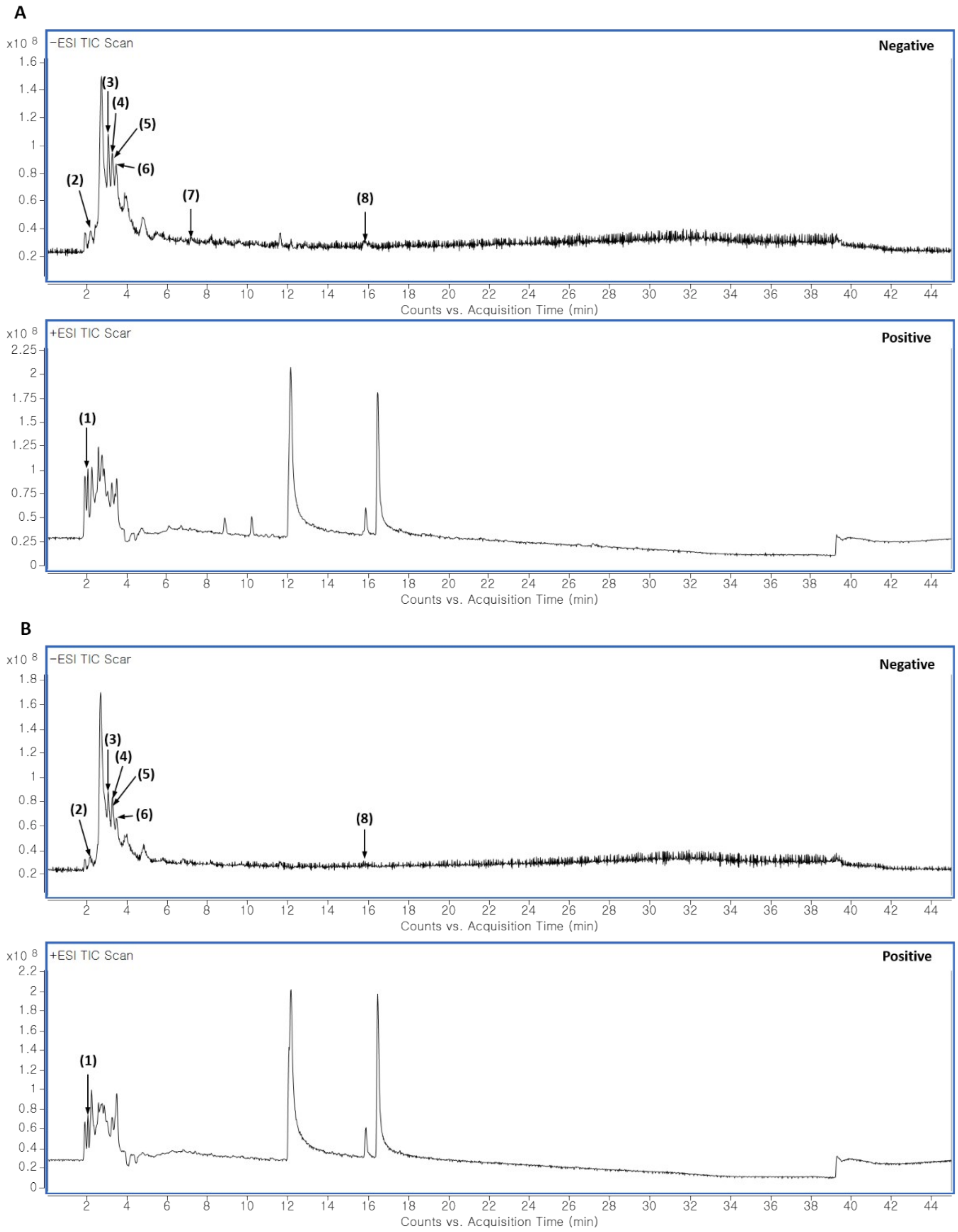

Fig. S3. LC-Q-TOF/MS chromatogram of *A. triphylla* root (AR) (A) and its hydrothermal extract (ARE) (B) in negative and positive ion modes. The eight identified differential metabolites are marked on the chromatograms. (1) Arginine, (2) Sucrose, (3) Quinic acid, (4) Citric acid, (5) Ascorbic acid, (6) Gallic acid, (7) Tangshenoside I, (8) Sanleng acid.

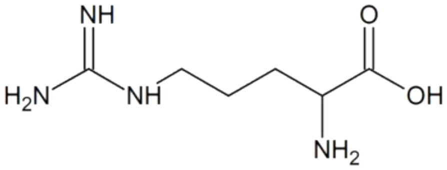

Arginine

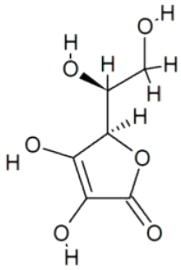

Ascorbic acid

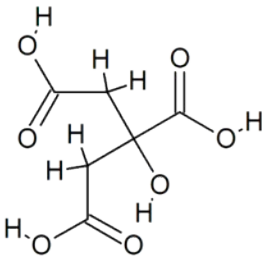

Citric acid

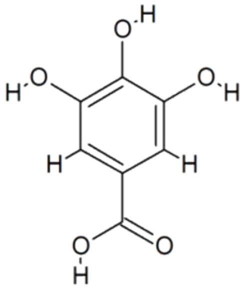

Gallic acid

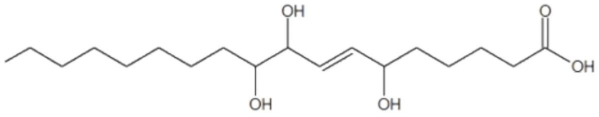

Sanleng acid

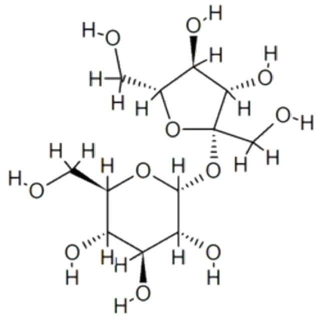

Sucrose

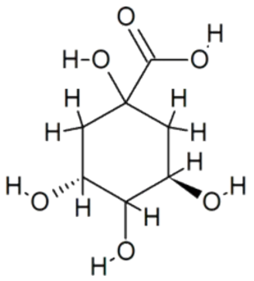

Quinic acid

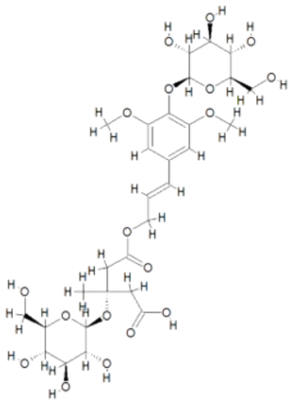

Tangshenoside I

Fig. S4. Chemical structures of the eight differential metabolites identified between *A. triphylla* root (AR) and its hydrothermal extract (ARE).
